# Prevalence of dynapenia and overlap with disability, depression, and executive dysfunction

**DOI:** 10.11606/s1518-8787.2023057004580

**Published:** 2023-07-18

**Authors:** Ivan Abdalla Teixeira, Evandro Silva Freire Coutinho, Valeska Marinho, Erico Castro-Costa, Andrea Camaz Deslandes

**Affiliations:** I Universidade Federal do Rio de Janeiro Instituto de Psiquiatria Rio de Janeiro RJ Brasil Universidade Federal do Rio de Janeiro, Instituto de Psiquiatria. Rio de Janeiro, RJ, Brasil; II Universidade do Estado do Rio de Janeiro Instituto de Medicina Social Rio de Janeiro RJ Brasil Universidade do Estado do Rio de Janeiro, Instituto de Medicina Social. Rio de Janeiro, RJ, Brasil; III Fundação Oswaldo Cruz Instituto René Rachou Belo Horizonte MG Brasil Fundação Oswaldo Cruz. Instituto René Rachou. Belo Horizonte, MG, Brasil.

**Keywords:** Activities of Daily Living, Depression

## Abstract

**OBJECTIVE:**

This study aims to investigate handgrip strength and dynapenia prevalence among older adults stratified by Brazilian macroregions. Additionally, we aim to evaluate the overlap between dynapenia and Instrumental Activities of Daily Living (IADL) disability, depression, and executive dysfunction on a national basis and by each Brazilian macroregion.

**METHODS:**

This cross-sectional analysis was based on data from the Brazilian Longitudinal Study of Aging (ELSI-Brazil). A multistage cluster sample design was used, with a representative population-based study of non-institutionalized community-dwelling Brazilians aged ≥ 50 years from 70 municipalities across all five macroregions of the country. The outcome variable was dynapenia. Covariables were IADL disability, depression, and executive dysfunction. The Brazilian macroregions were used for stratification. In addition, the following additional variables were included: age group, gender, education level, macroregions (North, Northeast, Southeast, South, and Midwest), self-reported health, multimorbidity, and falls.

**RESULTS:**

A total of 8,849 (94%) of the sample provided complete information for the handgrip strength assessment and were included in this analysis. Dynapenia prevalence was higher in North and Northeast regions (28.5% and 35.1%, respectively). We identified statistically significant differences between different macroregions for dynapenia, IADL disability, and verbal fluency, with worse values in the North and Northeast regions. In the North and Northeast macroregions, nearly half of the subjects that presented executive dysfunction and IADL disability also had dynapenia. There was a more significant overlap in the prevalence of all four conditions in the North and Northeast regions (4.8% and 5.5%, respectively), whereas the overlap was smaller in the South (2.3%). There was also a smaller overlap in the prevalence of dynapenia and depression in the South (5.8%) compared with other macroregions.

**CONCLUSIONS:**

Macroregions in Brazil exhibit marked differences in the prevalence of dynapenia and in its overlap with IADL disability, depression, and executive dysfunction.

## INTRODUCTION

Dynapenia is the age-related muscle strength loss^1^ that is partially explained by decreased muscle mass and has been associated with significant health outcomes in older adults, such as loss of mobility^2^, chronic diseases^3^, cognitive decline and depression^2,4^, falls and fractures^7^, cardiovascular events and hospitalization^8^, poor quality of life^9^, and death^2,10,11^. Consequently, the maintenance of muscle strength, more than muscle mass, during the aging process is a crucial public health concern.

Basic Activities of Daily Living (BADL)^12^ and Instrumental Activities of Daily Living (IADL)^13^) are influenced by dynapenia. The association of BADL with muscle strength and poor physical performance is easily understood^1^. In contrast, IADL depend on neural system integrity, also related to dynapenia^1^. The relationship between dynapenia and worse IADL is also observed among older adults with mild to moderate Alzheimer’s disease^16^. Additionally, dynapenia has been associated with depression and cognition^2,4,5^. Likewise, weakness is associated with worst cognitive performance in individuals with and without major depression^17^. Furthermore, executive dysfunction predicts cognitive decline^18^ and is a key contributor to IADL disability in patients with mild cognitive impairment and Alzheimer’s disease^19,20^, and is a common feature of cognitive dysfunction associated with depression^21^. These data suggest a complex relationship between dynapenia, IADL disability, mood, and cognition, with multiple effects in different clinical contexts.

Since dynapenia impacts older adults’ health and is associated with negative outcomes, determining the prevalence of dynapenia may help health systems minimize its consequences using proper interventions^22^. In this context, the Brazilian Longitudinal Study of Aging (ELSI-Brazil) revealed a high prevalence of dynapenia (17.2%) in older adults in Brazil^12^. However, handgrip strength is influenced by sociodemographic and lifestyle factors^25^ and dynapenia prevalence is expected to vary between the different populations. Furthermore, Brazil is a large country, with continental proportions and dramatic sociodemographic and health contrasts between its different macroregions. For example, North and Northeast regions present worse socioeconomic features and health indexes compared to South and Southeast^26,27^.

Therefore, comprehensive knowledge of dynapenia prevalence across Brazil’s macroregions, as well as the overlap of dynapenia with disability, depression, and executive dysfunction is crucial for establishing specific public health policies according to the region. Moreover, the efficient treatment of dynapenia may also facilitate promoting the intrinsic individual capacity and healthy aging. Thus, the objective of this study was: 1) to investigate handgrip strength and dynapenia prevalence of older adults stratified by all the different Brazilian macroregions; and 2) to evaluate dynapenia relationships between IADL disability, depression, and executive dysfunction on a national basis and by each Brazilian macroregion.

## METHODS

### Data Source

This is a cross-sectional analysis based on data from the first wave (baseline) of the Brazilian Longitudinal Study of Aging (ELSI-Brazil), conducted from 2015 to 2016^28^. The ELSI-Brazil is a representative population-based study of non-institutionalized community-dwelling Brazilians aged ≥ 50 years from 70 municipalities in the five macroregions of the country. No exclusion criteria were applied. A multistage cluster sample design was used. The urban and rural areas were stratified into small, medium, and large municipalities, according to the size of their population. In the first step of the sampling process, municipalities were selected within each stratum. The second step comprised the selection of a total of 176 census tracts from the previously selected municipalities. Then, in the third step, the households were obtained from these census tracts. All residents in the selected household were eligible if they were 50 years old or more. The planned sample size was 10,000 (9,412 participated), which should allow for an estimated prevalence of 1% (sample error = 0.25%), or a prevalence of 5% (sample error = 0.55%), at a level of significance of 95% and an effect sample design of 1.5. For inequality comparisons, it was possible to identify differences of 3.6% between the top and bottom quintiles, for a prevalence of 10% with a power test of 80%. Further details on sample design and methodology have been described previously^29^.

### Variables and Data Collection

After signing informed consent forms, all subjects were interviewed by a trained evaluator using a structured questionnaire, who also performed physical examination.

### Dynapenia

Dynapenia was defined by handgrip strength (*i.e.*, maximal voluntary force) measured as isometric strength using a hydraulic hand dynamometer (Saehan Corp., South Korea; Model SH5001). Cut-off values for dynapenia were < 27 kg for men and < 16kg for women^30,31^. In a sitting position, participants were instructed to grasp the dynamometer in their dominant hand, keeping their arm tight to the body with the elbow forming a 90° angle. Participants were then required to squeeze the device as hard as they could for 2 s. The test was performed three times, with a 1-min rest between each test. The mean value of the three trials was used as the isometric strength score.

### Disability

IADL disability was defined as difficulty in at least one of the following nine activities: taking care of self-hygiene, preparing a warm meal, handling money, using transportation, shopping, using the telephone, handling their medication, and performing light domestic tasks. BADL disability was defined as difficulty in at least one of the following activities: walking across a room, dressing, bathing, eating, getting in and out of bed (transferring), and toileting.

### Depression

Depressive symptoms were assessed using the eight-item version of the Centre for Epidemiological Studies Depression Scale (CES-D8 scale), an abridged version of the CES-D20. The CES-D8 score ≥ 4 corresponds to the traditional cut-off of 16 points on the CES-D20, which indicates a clinical diagnosis of depression^32^. Therefore, a dichotomized (yes/no) score was used as outcome measure. The CES-D8 is designed to assess the following symptoms exhibited by the patient in the previous week: depression, lack of happiness, loneliness, sadness, felt everything they did was an effort, restless sleep, inability to get going, and lack of energy. Each question is assigned one point, with a maximum possible score of eight.

### Executive Dysfunction

In this analysis, verbal fluency was selected as a proxy for executive dysfunction. Verbal fluency was determined using the semantic verbal fluency test (animal category). Participants were asked to say the name of as many animals as they could in a 1-min period.

Verbal fluency was chosen since it assesses executive function and its cutoffs have been validated for the Brazilian population stratified by levels of education^33^, reducing possible bias when comparing cognitive function between the different macroregions. The cut-off points were 9 for people with < 8 years of education and 13 for individuals with a higher level of education.

### Other Variables

Additional variables included in this analysis were: age group (50–59 years, 60–69 years, 70+ years), gender, education level (illiterate, 1–4 years, 5–8 years, 9–11 years, 12+ years), macroregions (North, Northeast, Southeast, South, and Midwest), self-reported health (poor, regular, good/very good), multimorbidity, and falls.

Multimorbidity was defined according to the number of the following self-reported chronic diseases: hypertension, diabetes, chronic obstructive pulmonary disease, osteoarthritis, stroke, asthma, cancer, renal disease or heart disease, depression, Parkinson’s disease, and Alzheimer’s disease. The history of falls and hospitalization was based on the 12 months before the interview.

### Statistical Analysis

First, the data was analyzed to identify the frequency and distribution of the variables. The means and proportions, with respective standard deviation and 95% confidence intervals, were calculated for the variables of interest across all the samples, stratified by macroregion. Second, the means and 95% confidence intervals were estimated for handgrip strength according to sociodemographic and health variables. Handgrip strength was also assessed for estimating the prevalence of dynapenia, using the cut-off values for men (< 27 kg) and women (< 16kg). The same analysis was conducted after the data were stratified for the five macroregions.

The statistical significances for the differences were calculated using chi-squared tests and One Way ANOVA. The *lincom* command of the Stata program was used to calculate point estimates, standard errors, z-statistics, p-values, and confidence intervals for linear combinations of the estimated parameters.

Venn diagrams were constructed to describe the overlap among dynapenia, IADL disability, depression, and executive dysfunction for the Brazilian macroregions.

All statistical analyses were conducted with the Stata 16.1 program, using the command *SVY* to consider the complex design of the sampling process.

### Ethics Statement

The ELSI-Brazil Project was approved by the Ethics Commission of Fiocruz, Minas Gerais, Brazil (CAAE 34649814.3.0000.5091). All participants signed separate informed consent forms for all research procedures.

## RESULTS

Of the 9,412 ELSI-Brazil baseline participants, 8,849 (94%) provided complete information for the handgrip strength assessment and were included in this analysis. The main characteristics of the participants are presented by region in Table 1. In the total sample, the average age was 62.2 years. The majority were women, had ≤ 4 years of schooling (38.4%), and reported regular health (44.8%). Approximately one-third had three or more chronic diseases and about one-fifth reported at least one fall in the 12 months before the study. IADL disability and low verbal fluency were present in approximately 40% of the participants, whereas depression was found in 32%. There were statistically significant differences between regions for the following variables: years of education, self-reported health, multimorbidity, IADL disability, and verbal fluency, with worse values for the North and Northeast compared with the other regions, except for multimorbidity.

**Table 1:** Characteristics of the participants by region.

Characteristic	Total	North	Northeast	Southeast	South	Midwest	X^2^/F	p-value
					
	n = 8,849	n = 721	n = 2,336	n = 3,676	n = 1,224	n = 892		
Age^a^ - average (95%CI)	62.2 (61.4–63)	61.5 (60.2–63)	62.2 (60.8–63.7)	62.3 (61–63.7)	62.3 (60.4–64.1)	61.5 (59.3–63.9)	0.3	0.89
Gender: Women^b^ - % (95%CI)	53.3 (50.4–56.2)	50.1 (42.3–58)	53.7 (47.6–59.7)	53.8 (49.5–58)	52.9 (45–60.8)	52.1 (41.6–62.4)	3	0.97
Education (years)^b^ - %(95%CI)							497.1	< 0.001
12 or more	8.1 (6.9–9.4)	7.4 (4.4–12.2)	5.5 (3.6–8.2)	8.9 (7–11.3)	9.8 (7–13.5)	6.9 (4.8–9.8)		
9–11	18.6 (16.8–20.5)	23.9 (14–37.8)	13.7 (12–17)	20.2 (18–23.4)	18.2 (13–25.1)	20.9 (15.1–28.2)		
5–8	22.0 (19.8–24.5)	25.5 (18.3–34.4)	16.9 (14–20.2)	22.9 (20.4–25.6)	26.1 (18–36.3)	21.2 (16–27.6)		
1–4	38.4 36.1–40.8)	28.1 (18.9–39.6)	38.3 (34.2–42.7)	39.3 (36–42.8)	39.8 (31.7–48.5)	37.7 (31.2–44.7)		
Iliterate	12.7 (10.4–15.4)	14.9 (7–28.8)	25.4 (20.5–31)	8.5 (6.4–11.3)	6 (3.7–9.5)	13.1 (7.8–21.3)		
Self-reported health^b^ - % (95%CI)							189.2	< 0.001
Good/very good	43.9 (41.4–46.4)	32.4 (29.4–35.7)	34.0 (31.1–36.9)	49.4 (45.4–53.3)	48.5 (42.8–54.1)	38.3 (32.4–44.5)		
Regular	44.8 (43–46.7)	51.9 (47.7–56.1)	51.9 (49.3–54.3)	41.4 (38.5–44.3)	40.6 (36.1–45.3)	49.3 (46.7–51.9)		
Poor	11.2 (10–12.4)	15.5 (12.9–18.7)	14.1 (12–16.5)	9.2 (7.7 – 10.9)	10.8 (8.8–13.3)	12.3 (7.9–18.8)		
Multimorbidity^b^ - % (95%CI)							65.5	0.002
0–1	45.1 (42.7–47.5)	48.7 (41.3–56.1)	51.0 (48.7–53.3)	44 (40–47.9)	39.7 (34.7–44.8)	42.4 (37.8–47.2)		
2	22.2 (21.2–23.2)	21.3 (17.5–25.7)	21.9 (20.4–23.6)	21.9 (20.5–23.4)	22.9 (19.9–26.3)	23.8 (20.4–27.5)		
3 or more	32.7 (30.5–34.8)	30 (24.5–36)	27 (25.1–28.9)	34.0 (30.5–37.7)	37.3 (33.6–41.2)	33.7 (27.1–41)		
Falls^b^ - % (CI 95%)	21.9 (20.6–23.2)	19.4 (17.5–21.4)	22.6 (20.6–24.6)	21.9 (19.7–24.3)	20.9 (18.9–23)	23.9 (19.6–28.7)	4.7	0.47
BADL disability^b^ - % (95%CI)	14.6 (13.3–15.9)	14.0 (10.2–18.8)	16.3 (14.4–18.3)	13.8 (11.6–16.3)	14.7 (12.3–17.6)	14.0 (10.4–18.6)	7.2	0.55
IADL disability^b^ - % (95%CI)	39.2 (36.2–42.3)	44.7 (38.7–50.9)	47.7 (43.1–52.3)	35.7 (30.8–41.0)	35.4 (28.6–42.8)	38.8 (27.5–51.4)	98.8	0.01
Verbal fluency^a^ - average (95%CI)	12 (11.7–12.4)	11.4 (10.8–12)	10.8 (10.1–11.5)	12.4 (12–12.8)	12.6 (11.8–13.4)	13.1 (12.3–13.9)	6.8	< 0.001
Low verbal fluency^b^ - % (95%CI)	40.2 (38.0–42.5)	48.4 (44.2–52.7)	47.9 (42.2–53.6)	38.2 (35.7–40.8)	34.7 (29.7–40.0)	34.7 (31.2–38.4)	97.7	0.001
CES-D8^a^ - average (95%CI)	3.1 (3–3.2)	3.1 (3–3.2)	3.2 (3.1–3.4)	3.1 (3–3.2)	2.9 (2.8–3.1)	3.3 (2.9–3.6)	1.6	0.17
Depression^b^ - % (95%CI)	31.6 (30.0–33.2)	31.7 (29.3–34.2)	34.3 (31.2–37.6)	30.7 (31.2–37.6)	28.9 (24.5–33.7)	35 (28.7–41.8)	14.7	0.16

CI: Confidence Interval. BADL: Basic Activities of Daily Living. IADL: Instrumental Activities of Daily Living. CES-D8: the eight-item version of the Centre for Epidemiological Studies Depression Scale. a: variables analyzed using ANOVA. b: variables analyzed using chi-squared test.

**Figure 1:**
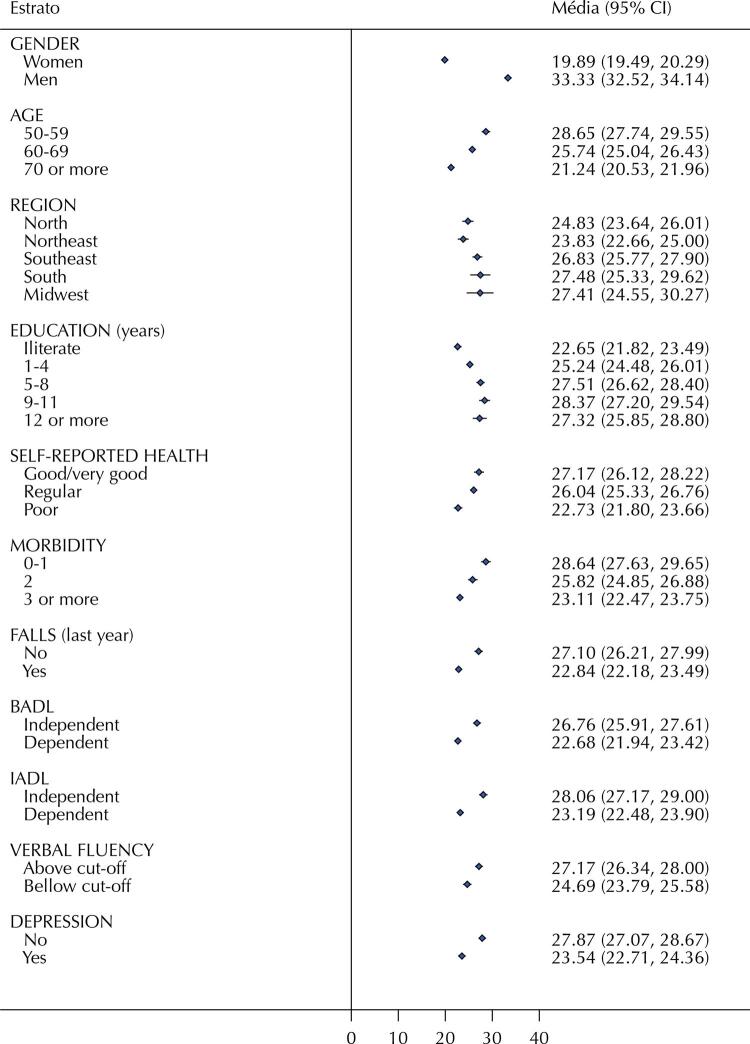
Handgrip strength values (mean and 95% confidence interval) stratified by sociodemographic, macroregions, and health variables.

**Figure 2:**
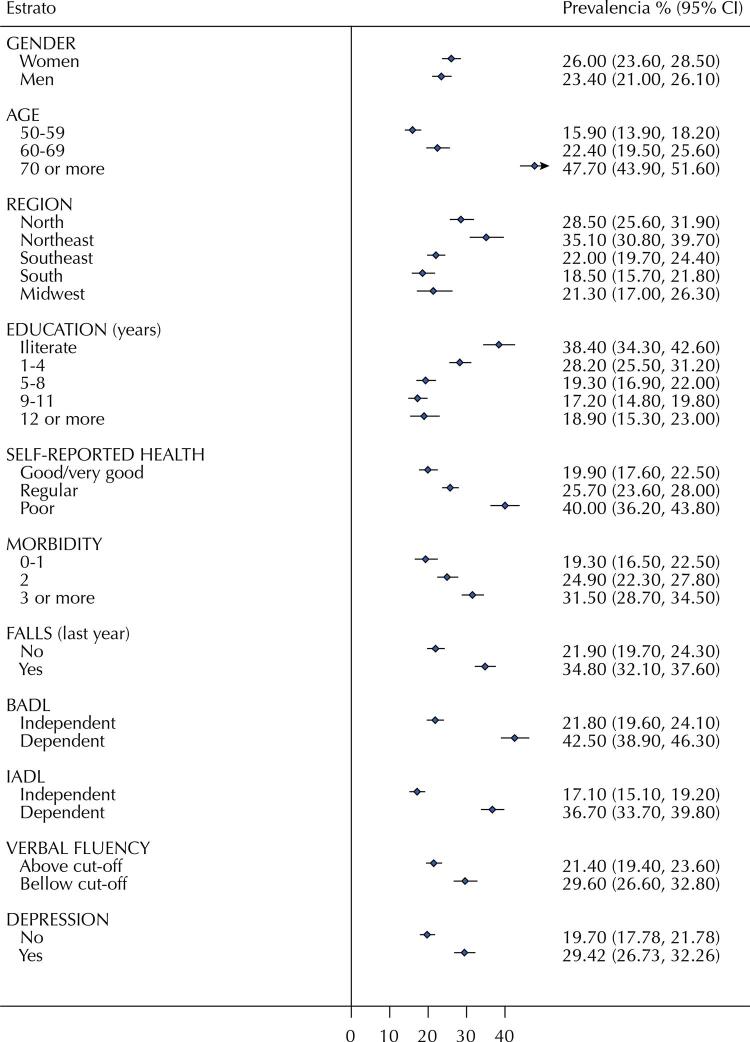
Dynapenia prevalence (% and 95% confidence interval) stratified by sociodemographic, macroregions, and health variables.

Figures 1 and 2 show the handgrip strength values (mean and 95% confidence interval) and dynapenia prevalence (% and 95% confidence interval), respectively. These data were stratified by sociodemographic, macroregion and health variables. Residents in the North and Northeast regions tend to show lower handgrip strength values when compared to the other three macroregions (p-values ≤ 0.04). Consistent with this finding, the highest prevalence of dynapenia was observed in North and Northeast macroregions (p-values ≤ 0.02). Handgrip strength values were also lower in these macroregions, with a higher prevalence of dynapenia among older adults (70+ years; p < 0.001), those with a lower level of education (≤ 4 years of education and illiterate; p < 0.001), worse self-reported health (p < 0.001), higher prevalence of comorbidities (p < 0.001), history of falls (p < 0.001), and BADL or IADL disability (p < 0.001 for both). Dynapenia was more prevalent among individuals who exhibited poor performance in verbal fluency tests and reported symptoms of depression, compared to those without these conditions. P-values were less than 0.001 for these two variables.

BADL: Basic Activities of Daily Living. IADL: Instrumental Activities of Daily Living.

BADL: Basic Activities of Daily Living. IADL: Instrumental Activities of Daily Living.

The total prevalence of dynapenia was 24.8% (95%CI: 22.7%–27.1%). Table 2 shows the prevalence and 95% confidence interval of dynapenia stratified by the sociodemographic and health variables, compared between all macroregions. The prevalence of dynapenia was higher in the North and Northeast regions and lower in the South independently of sociodemographic or health conditions.

**Table 2:** Dynapenia prevalence per macroregion, stratified by the sociodemographic and health variables.

Characteristic	North	Northeast	Southeast	South	Midwest
Gender - % (95%CI)					
Women	30.4 (26.1–35.1)	35.2 (29.7–41.2)	19.7 (15.4–24.8)	23.6 (21.0–26.4)	21.9 (18.8–25.4)
Men	26.9 (20.0–35.1)	34.9 (29.9–40.1)	17.2 (14.4–20.4)	20 (17–23.3)	20.5 (12.6–31.7)
Age - % (95%CI)					
50–59	18.6 (15.6–22.0)	24.7 (19.6–30.7)	9.9 (7.1–13.6)	14 (12.1–16.2)	11.5 (8.0–16.3)
60–69	27.1 (21.8–33.1)	34.2 (27.9–41.2)	14.6 (12.3–17.2)	18.9 (15.3–23.2)	21.2 (14.1–30.6)
70 or more	59.2 (52.3–65.9)	58.7 (48.8–67.8)	43 (33.2–53.3)	42.9 (38.5–47.4)	46.8 (40.8–52.9)
Education (years) - % (95%CI)					
12 or more	17.1 (7.5–34.5)	33.7 (25.1–43.5)	7.8 (3.9–14.9)	19.1 (14.8–23.3)	16.6 (8.5–29.8)
9–11	23.7 (19.8–28.1)	26.1 (10.6–32.6)	13.6 (7.3–23.9)	15.2 (12–19.2)	11.1 (7.8–15.4)
5–8	16.7 (13.2–20.9)	31.0 (25.3–37.2)	15.7 (10.9–22.1)	17.9 (14.8–21.4)	11.8 (7.8–17.5)
1–4	36.2 (26.6–47.1)	34.3 (27.3–42.1)	22.9 (17.4–29.4)	26.6 (23.5–29.9)	28 (18.4–40.1)
Iliterate	47 (40.0–54.1)	43.9 (38.8–49.1)	33.9 (17.7–54.9)	30.2 (24.2–37.1)	34.7 (31.8–37.7)
Self-reported health - % (95%CI)					
Good/very good	27 (20.6–34.5)	33 (28–38.3)	12.8 (9.1–17.8)	17.7 (15.1–20)	17.1 (12.7–22.7)
Regular	25.2 (20.8–30.2)	33 (28.4–37.9)	19.8 (16.2–24)	24 (21.2–27)	21.9 (17.6–27.1)
Poor	43.8 (37.4–50.5)	47.5 (39.3–55.9)	39.1 (30.7–48.1)	35.5 (30.6–40.6)	31.3 (23.5–40.4)
Morbidity - % (95%CI)					
0–1	22 (15.2–30.9)	30.2 (25–35.9)	9.8 (7.3–13)	16 (12.7–20)	16 (11.6–21.7)
2	28.0 (22.2–34.6)	36.4 (31.1–42.1)	19.8 (14.9–25.7)	21.5 (17.8–25.8)	19.2 (13.8–26.0)
3 or more	37.8 (32.6–43.3)	42.7 (35.7–50)	26.5 (21.6–32)	28.9 (24.9–33.2)	27.8 (22.6–33.8)
Falls - % (95%CI)					
No	24.9 (20.8–29.5)	32.7 (28.1–37.8)	15.1 (11.7–19.2)	19.1 (16.8–21.7)	19.0 (13.8–25.6)
Yes	44.2 (36.5–52.2)	42.9 (37.1–48.9)	31.6 (26.2–37.5)	31.7 (28.2–35.5)	28.0 (23.4–33.0)
BADL - % (95%CI)					
Independent	26.4 (23.1–30.1)	31.6 (26.8–36.8)	14.8 11.6–18.7)	19.3 (16.9–22)	18.4 (14.2–23.6)
Dependent	42.2 (30.9–54.3)	52.87 (45.4–60.1)	39.9 (32.8–47.5)	38.1 (32.8–43.6)	38.3 (29.7–47.7)
IADL - % (95%CI)					
Independent	18.5 (12.9–25.8)	26.1 (21.3–31.6)	11.3 (7.6–16.4)	15.8 (13.6–18.3)	13.3 (9.8–17.8)
Dependent	40.8 (33.1–48.9)	44.9 (39.4–50.6)	31.7 (23.6–41.2)	32.8 (29.2–36.6)	33.7 (27.2–40.9)
Verbal fluency - % (95%CI)					
Above cut-off	26.3 (22.0–31.1)	31.8 (27.8–36)	16.3 (13.7–19.4)	19.1 (16.5–22)	17.2 (13.5–21.8)
Bellow cut-off	30.7 (26.4–35.5)	38.4 (31.5–45.8)	22.5 (17.7–28.3)	25.4 (23.4–29.6)	28.4 (19.5–39.3)
Depression - % (95%CI)					
No	26.1 (20.6–32.5)	28.6 (23.9–33.9)	13.6 (10.9–16.8)	17.5 (15.6–19.6)	15.3 (9.2–24.3)
Yes	31.2 (23.9–39.6)	36.6 (31.4–42.1)	20.1 (15.9–25.1)	28.4 (24.3–32.9)	27.8 (23.4–32.5)

CI: Confidence interval. BADL: Basic Activities of Daily Living. IADL: Instrumental Activities of Daily Living.

**Figure 3:**
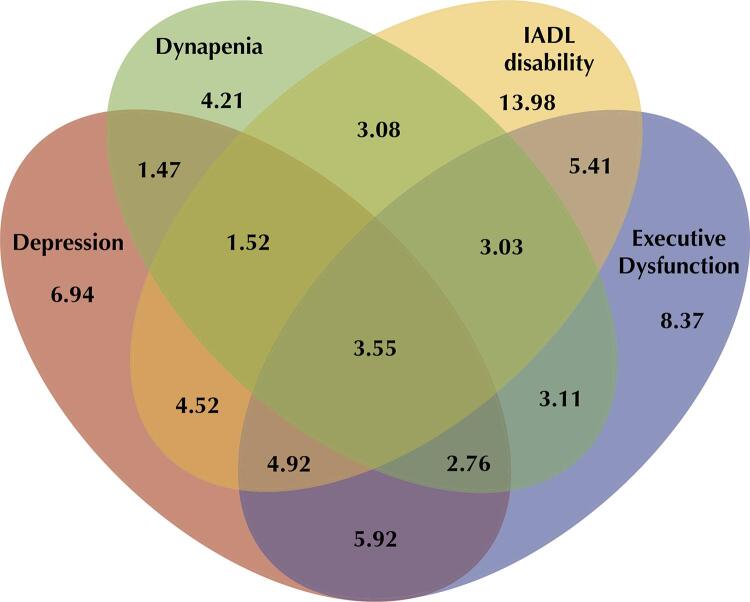
Venn diagram, Brazil.

The Venn diagram in Figure 3 shows the overlap prevalence between dynapenia, IADL disability, depression, and executive dysfunction in Brazil. The prevalence of dynapenia alone (4.2%) was lower than for dynapenia overlapped with at least one other variable (18.5%). The same pattern was observed for IADL disability (14.0% *vs*. 26.0%), depression (6.9% *vs*. 27.4%), and executive dysfunction (8.4% *vs*. 28.7%). The prevalence of dynapenia overlap with IADL disability was greater when executive dysfunction, depression, or both were present compared with dynapenia alone (8.1% *vs*. 3.1%). The prevalence of executive dysfunction overlap with IADL disability was estimated at 16.9%, being 6.6% for participants who also have dynapenia.

IADL: Instrumental Activities of Daily Living. n = 7915.

**Figure 4:**
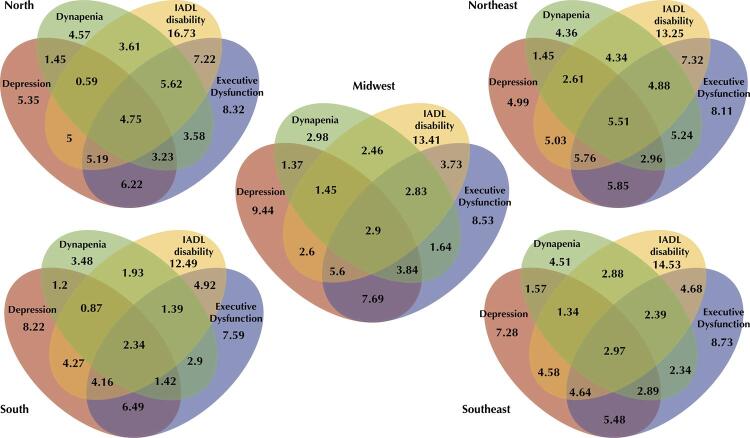
Venn diagram by macroregions.

The Venn diagrams in Figure 4 show the overlap prevalence between dynapenia, IADL disability, depression, and executive dysfunction by each macroregion. Interestingly, the overlap in the prevalence of all four conditions was more significant in the North and Northeast regions (4.7% and 5.5%, respectively) compared with that in the South (2.3%). In addition, the prevalence of IADL disability alone was higher in the North than in the other regions, whereas the prevalence of depression alone and dynapenia alone was higher in Midwest and South regions. Regarding the overlap in executive dysfunction and IADL disability, a higher prevalence was observed in the North (22.8%) and Northeast (23.5%), with a lower prevalence in the South (12.8%), followed by the Southeast (14.7%) and Midwest (15.1%). Nearly half of the subjects presenting executive dysfunction and IADL disability in the North and Northeast macroregions also had dynapenia (10.4% with dynapenia *vs*. 12.4% without dynapenia in the North and 10.4% *vs*. 13.1% in the Northeast). In contrast, this proportion was smaller in the other macroregions (South, 3.7% *vs*. 9.1%; Southeast, 5.4% *vs*. 9.3%; Midwest, 5.7% *vs*. 9.3%). There was also a lower prevalence of dynapenia overlap with depression in the South (5.8%) than in the other macroregions (North 9.0%, Northeast 12.5%, Southeast 8.8%, and Midwest 9.6%). Finally, the prevalence of dynapenia overlap with IADL disability was higher in the North (14.6%) and Northeast (17.3%) compared to that in the South (6.5%), with intermediate prevalence in the Southeast and Midwest (9.6%).

IADL: Instrumental Activities of Daily Living.

## DISCUSSION

To our acknowledgment, this is the first study to evaluate the prevalence of dynapenia across all Brazilian macroregions and explore the overlaps between dynapenia, IADL disability, depression, and executive dysfunction. We verified variations in the prevalence of dynapenia between macroregions, and found significantly higher prevalence in the North and Northeast, lower prevalence in the South and Southeast, and intermediate prevalence in the Midwest. These findings support previous reports that handgrip strength is influenced by socioeconomic factors, such as poor health, malnutrition, and low level of physical activity. These factors tend to be more common in less developed areas with poorer access to the health system, which results in lower muscle mass and function among the population^25^. Following this, the participants in the North and Northeast also presented lower educational levels and self-reported health in our study. Furthermore, other national data show that these regions are also ranked lower on the Human Development Index than the other regions, whereas the Southeast, South, and Midwest present similar indexes^27^. Moreover, a previous ELSI study also showed that lower levels of education and physical activity increased the odds of having dynapenia, accounting for 10.0% and 16.7% of the population attributable fractions^12^, respectively. Since weakness has been associated with both disability^12^ and mortality^10,11,35^, it is also possible that the higher prevalence of dynapenia in the North and Northeast regions contributes to the lower healthy life expectancy observed in these regions^34^.

Interestingly, dynapenia is also more prevalent among middle-aged adults (50–59 years) in the North and Northeast regions, suggesting that inequalities in the aging process and disparities across the age groups are implicated in the higher prevalence of dynapenia in these macroregions. Consequently, individuals in the North and Northeast regions of Brazil are at a disadvantage since their middle age, highlighting the necessity of early intervention to target the causes of dynapenia, especially in less developed areas. Similarly, other studies confirm the relationship between dynapenia and socioeconomic status in early and adult life. For example, the Survey of Health, Ageing, and Retirement in Europe (SHARE) in individuals aged ≥ 50 years living in 14 European countries showed that older adults with socioeconomic disadvantages and unhealthy lifestyles in early- and adult-life had a greater risk of low muscle strength^36^.

Handgrip cutoffs are essential to guarantee correct estimation of the prevalence of dynapenia prevalence and better understand the relationship between muscle strength and other factors. In the absence of a Brazilian consensus, the handgrip cut-off proposed by European sarcopenia consensus has been applied in other dynapenia and sarcopenia surveys in Brazil^12,37^. Although normative data on handgrip strength among Brazilian older people has been recently published^42^, some issues were considered to not chose those proposed cut-offs. First, this study was based on the Frailty in Brazilian Older People data from different surveys that were mainly performed in urban areas, which may be a limitation when analysing a more widely distributed population as our sample. Second, differently from most of the literature in dynapenia and sarcopenia, the proposed handgrip cut-offs are stratified by height and age. This may be interesting to better compare handgrip strength between individuals, but it also may underdiagnose weakness among the oldest adults since it is known that age is an important risk factor to the development of dynapenia and sarcopenia. In line with previous data, our study confirms the utility of the handgrip cut-off that was proposed by the European sarcopenia consensus in a large Brazilian population sample. This cut-off was used to equalize the differences observed in crude handgrip measures between both genders and to highlight the expected relationships of dynapenia with age, level of education, self-reported health, morbidity, and falls in all the Brazilian macroregions.

As suggested by previous reports^13^, our study showed that dynapenia is associated with BADL and IADL disability. Thus, both IADL and BADL disability appear to have a more significant relationship with the prevalence of dynapenia than with executive dysfunction and depression, as shown in Figure 1b. Figures 2a and 2b show that most older adults with dynapenia have IADL disability, depression, or executive dysfunction. Therefore, the higher prevalence of IADL disability in the North and Northeast regions may be partially explained by the similarly higher prevalence of dynapenia and executive dysfunction, but not by depression, which showed similar prevalence in all macroregions. However, a significant proportion of older adults with IADL disability have none of these conditions, especially in the North region, suggesting that other variables, such as multimorbidity, may also contribute to IADL disability in Brazil.

Studies have shown a relationship between muscle strength and depression^4,5^, and that depression is more prevalent in older adults with a history of dynapenia^2^. In this study, depression was also associated with dynapenia. Figure 2b demonstrates a higher prevalence of depression alone (without the other three variables) in the South and Midwest, with lower prevalence in the North and Northeast. These data indicate the higher prevalence of depression in the North and Northeast as a comorbid with dynapenia, IADL disability, and executive dysfunction. The prevalence of depression among older adults in our study was consistent with that reported previously^43^. Although lower prevalence has also been reported^46,47^, these studies were conducted in urban centers^46^ or rural areas with specific colonization profiles (Italian immigrants)^47^, which may explain this difference.

Changes in cognition and grip strength occur in conjunction in later life^48^ and handgrip strength predicts cognitive decline^49^. Although few studies have been conducted in low and middle-income countries (LMIC), such as Brazil, our analysis demonstrates this association’s existence. In contrast to depression, but consistent with dynapenia, the prevalence of executive dysfunction was significantly higher in the North and Northeast. Although the prevalence of executive dysfunction alone was very similar between macroregions, we also observed a higher prevalence of executive dysfunction overlapping with IADL disability in the North and Northeast, with a lower prevalence in the South. Cognitive decline associated with IADL disability is the key definition of dementia. In this context, our study indicates an estimated prevalence of dementia in Brazil of 16.9% among older adults, being higher in the North and Northeast macroregions. Although it was not possible to confirm the diagnosis of dementia, this is an essential finding since other representative data on the prevalence of dementia in Brazil are still lacking^50^. Furthermore, similar to other LMICs, it is expected that the rate of underdiagnosis of dementia is higher in Brazil than that in high-income countries^51^. Another important finding in this scenario is that dynapenia is present in one-third to half of the individuals with both executive dysfunction and IADL disability, depending on the macroregion, thus demonstrating that dynapenia is a very common condition among individuals with probable dementia.

Analysis of the overlaps in the prevalence of dynapenia, IADL disability, low verbal fluency, and depression in Brazil and in its macroregions revealed that most the older adults presenting one of these four conditions also concomitantly present at least one other condition. Only 33.5% of the participants with one condition did not have another. Moreover, overlap between the four variables is three times greater than what would be expected only by hazard (3.5% *vs*. 1.2%). These findings corroborate that there may be an interaction between these conditions. However, a greater overlap between all four conditions was observed in the North and Northeast regions, whereas the overlap in the South region was smaller, suggesting that other factors may also contribute to these relationships.

Limitations of this study include the cross-sectional design, which precludes conclusions relating to causality. In addition, cognitive function was evaluated only by verbal fluency, which predominantly reflects executive function and operational memory. Nevertheless, more comprehensive neuropsychological tests would require specialized staff and longer interviews.

## CONCLUSION

The macroregions of Brazil present marked disparities in the prevalence of dynapenia, with higher prevalence in the North and Northeast regions, especially compared to the South. The relationships between dynapenia, IADL disability, depression, and executive dysfunction also vary between regions. Therefore, public health policies addressing dynapenia and IADL disability prevention should consider local characteristics. Dynapenia is associated with IADL disability, depression, and executive dysfunction across the country and the overlaps of these conditions are not the exception, but rather the rule. Interventional studies targeting dynapenia among individuals with depression, executive dysfunction, or both, are urgently required to prevent disability in older adults.
